# Adipose-derived endothelial and mesenchymal stem cells enhance vascular network formation on three-dimensional constructs in vitro

**DOI:** 10.1186/s13287-015-0251-6

**Published:** 2016-01-11

**Authors:** Alina Freiman, Yulia Shandalov, Dekel Rozenfeld, Erez Shor, Sofia Segal, Dror Ben-David, Shai Meretzki, Dana Egozi, Shulamit Levenberg

**Affiliations:** Inter-departmental Program in Biotechnology, Technion-Israel Institute of Technology, Haifa, 32000 Israel; Biomedical Engineering Department, Technion-Israel Institute of Technology, Haifa, 32000 Israel; Bonus BioGroup Ltd., Haifa, 3508501 Israel; Department of Plastic and Reconstructive Surgery, Kaplan Hospital, Rehovot, Israel

**Keywords:** Adipose MSCs, Microvascular ECs, Vascularization, PLLA/PLGA scaffolds, Tissue engineering

## Abstract

**Background:**

Adipose-derived mesenchymal stem cells (MSCs) have been gaining fame mainly due to their vast clinical potential, simple isolation methods and minimal donor site morbidity. Adipose-derived MSCs and microvascular endothelial cells have been shown to bear angiogenic and vasculogenic capabilities. We hypothesized that co-culture of human adipose-derived MSCs with human adipose-derived microvascular endothelial cells (HAMECs) will serve as an effective cell pair to induce angiogenesis and vessel-like network formation in three-dimensional scaffolds in vitro.

**Methods:**

HAMECs or human umbilical vein endothelial cells (HUVECs) were co-cultured on scaffolds with either MSCs or human neonatal dermal fibroblasts. Cells were immunofluorescently stained within the scaffolds at different time points post-seeding. Various analyses were performed to determine vessel length, complexity and degree of maturity.

**Results:**

The HAMEC:MSC combination yielded the most organized and complex vascular elements within scaffolds, and in the shortest period of time, when compared to the other tested cell combinations. These differences were manifested by higher network complexity, more tube alignment and higher α-smooth muscle actin expression. Moreover, these generated microvessels further matured and developed during the 14-day incubation period within the three-dimensional microenvironment.

**Conclusions:**

These data demonstrate optimal vascular network formation upon co-culture of microvascular endothelial cells and adipose-derived MSCs in vitro and constitute a significant step in appreciation of the potential of microvascular endothelial cells and MSCs in different tissue engineering applications that can also be advantageous in in vivo studies.

**Electronic supplementary material:**

The online version of this article (doi:10.1186/s13287-015-0251-6) contains supplementary material, which is available to authorized users.

## Background

For any engineered tissue, vasculogenesis and angiogenesis are crucial for integration and survival in vivo [1]. Inadequate vascular supply of the engineered tissue will prevent its assimilation with the host tissue, eventually leading to its deterioration [2]. Incorporation of bioengineered microvessels in cell-seeded tissue constructs presents a means of ensuring appropriate oxygen and nutrient exchange and can facilitate graft integration in vivo [3]. Optimal bioengineered vasculature can be obtained by co-culturing vascular endothelial cells (ECs) and perivascular cells on three-dimensional (3D) poly-L-lactic acid (PLLA)/poly-lactic-co-glycolic acid (PLGA) constructs [2, 4].

Adipose-derived stem cells comprise one of the most promising stem cell populations identified thus far [5], primarily due to the ubiquity of adipose tissue, simple isolation techniques, and minimal donor site morbidity and patient discomfort [6, 7]. Due to the capacity of its mesenchymal stem cell (MSC) content to differentiate to a variety of cell types of the mesodermal lineage [8, 9], recent tissue engineering applications have integrated adipose-derived MSCs to achieve regeneration of a variety of tissue types, including vascular tissues [10, 11]. Many studies have demonstrated vascular formation and maturation in vitro, mediated by angiogenic factors secreted by MSCs supporting cultured ECs [10, 12]. The origin of ECs selected for vascular formation applications has been suggested to be very important as well, where adipose-derived microvascular ECs have been shown to display stronger angiogenic capacities, when compared to those derived from macrovessels [13, 14].

Our earlier studies have documented generation of vascular networks within porous biodegradable PLLA/PLGA constructs embedded with a co-culture of human umbilical vein endothelial cells (HUVECs) and human neonatal dermal fibroblasts (HNDFs) [2, 4, 15]. The present study aimed to monitor vascularization dynamics within 3D PLLA/PLGA scaffolds embedded with ECs isolated from either micro- or macrovessels, and to characterize the impact of supporting MSCs or HNDFs on the recorded vessel formation dynamics. We show that adipose-derived ECs supported by MSCs generate more developed vascular networks within 3D constructs and at a faster rate than other studied cell combinations. The possibility of using autologous adipose-derived MSCs [16] combined with microvascular ECs will both improve the formation rate and quality of vascular networks and will advance our abilities and potential to treat large tissue defects [17].

## Methods

### Cell culture

Adipose-derived MSCs (Bonus BioGroup Ltd., Israel) were cultivated in Dulbecco’s modified Eagle’s medium (DMEM; Gibco Life Technologies) supplemented with 10 % fetal bovine serum (FBS; European-grade, Biological Industries) and 1 % Glutamax (Gibco Life Technologies), for up to five passages. Human adipose microvascular endothelial cells (HAMECs; ScienceCell) isolated from human adipose tissue were cultivated in endothelial cell medium (ScienceCell) supplemented with 5 % FBS (ScienceCell) and endothelial cell growth supplement (ScienceCell), and harvested for the experiments during passages 4–8. HUVECs (Lonza) were grown in EGM-2 medium supplemented with endothelial cell growth medium BulletKit®-2 (EGM-2® BulletKit®, Lonza) and harvested for the experiments during passages 4–8. Human foreskin fibroblast cells (HNDFs; Lonza) were cultured in DMEM (Gibco Life Technologies) supplemented with 10 % FBS (Hyclone; Thermo Fisher Scientific), 1 % nonessential amino acids, and 0.2 % β-mercaptoethanol (Sigma-Aldrich). All cells were cultured in a 5 % CO_2_ humidified incubator at 37 °C.

### PLLA/PLGA scaffolds

3D porous biodegradable scaffolds were fabricated utilizing a salt-leaching technique with salt with a diameter of 212–600 μm, as previously described [18]. This range of NaCl particle sizes was used to allow formation of a sufficiently large interconnected pore-size network. At the same time, the pore size is sufficiently small to maintain optimal mechanical strength. The scaffolds were prepared from a 50 % PLLA (Polysciences) and 50 % PLGA (Boehringer Ingelheim) solution in chloroform. The polymer solution (0.24 ml) was added to sodium chloride particles (0.4 g) maintained in Teflon molds. The chloroform was allowed to evaporate overnight, and the salt was then leached out using distilled water, resulting in interconnected porous 3D scaffolds. The resulting 1 mm height scaffolds were cut out using a 6 mm diameter puncher to obtain 28.26 mm^3^ pieces, which were then soaked in 70 % ethanol (v/v) for 30 min, and washed three times in phosphate-buffered saline (PBS) for 5 min before use.

### Cultivation of cell combinations on PLLA/PLGA scaffolds

Four cell combinations were examined:

- HAMECs:MSCs
- HAMECs:HNDFs
- HUVECs:MSCs
- HUVECs:HNDFs

Our earlier studies established an optimal ratio of 5:1 between ECs and supporting cells (e.g., MSCs and HNDFs) [19]. In the present study, this ratio was maintained by seeding 0.25 × 10^6^ ECs and 0.05 × 10^6^ supporting cells per scaffold. First, the cells were mixed with 5 μl thrombin solution (Johnson & Johnson Medical, Israel). The same volume of a human fibrinogen solution (Johnson & Johnson Medical) was added to the cell-thrombin mixture, followed by rapid pipetting and seeding upon the PLLA/PLGA scaffolds, which were then placed on a non-tissue culture six-well plate dish. After the scaffolds were incubated in a highly humidified 37 °C, 5 % CO_2_ incubator for 30 min, 2 ml of EC medium, mixed with 2 ml of respective supporting cell medium (1:1 ratio), were added. Medium was changed every other day.

### Immunofluorescent staining of whole-mount scaffolds

Whole scaffolds were fixated in 4 % paraformaldehyde (Electron Microscopy Sciences) for 20 min, and then washed several times with PBS. Next, the scaffolds were treated with 0.3 % Triton X-100 (Bio Lab Ltd) for 10 min in order to permeabilize the cells. The scaffolds were washed with PBS and soaked in blocking solution (10 % FBS (v/v), 0.1 % Triton (v/v), 1 % glycine (w/v)) for 3 h at room temperature. Subsequently, samples were incubated overnight at 4 °C with the following primary antibodies (diluted in blocking solution): monoclonal mouse anti-human CD31 (1:50, Cell Marque) and monoclonal rabbit anti-α-smooth muscle actin (α-SMA) (1:200, Dako). After extensive washing, secondary antibodies (diluted in PBS) were added and incubated with the sample for 3 h at room temperature: Cy3-conjugated anti-mouse IgG (1:100, Jackson Immuno-research laboratory, PA) and Alexa 488-conjugated anti-rabbit IgG (1:400, Molecular Probes). The scaffolds were then stored in PBS until imaging.

### Immunofluorescent staining of paraffin-embedded sections

Whole scaffolds were cut perpendicularly to their horizontal plane (as illustrated in Additional file 1). Several slices were randomly chosen from the different areas of the scaffold to provide authentic data for scoring and analysis. Scaffolds were embedded in paraffin, using a standard fixation and embedding protocol, and then cut into 5-μm thick slices and placed on slides for immunofluorescent staining. Slides were incubated in a 60 °C heater for 30 min to dissolve the paraffin. Next, the slides were incubated at 95 °C for 20 min in Reveal Docloaker 10X (Biocare Medical) in a pressure cooker for epitope retrieval. After cooling, the slices were submerged in a 5 % bovine serum albumin (BSA) solution (w/v, Millipore) for 30 min. Subsequently, primary antibodies diluted in 5 % BSA solution were applied and incubated at 4 °C overnight: monoclonal mouse anti-human CD31 (1:50, Cell Marque) and monoclonal rabbit anti-α-SMA (1:200, Dako). Sections were washed three times in PBS for 5 min before being incubated with the following secondary antibodies: Cy3-conjugated anti-mouse IgG (1:100, Jackson Immuno-research laboratory) and Alexa 488-conjugated anti-rabbit IgG (1:400, Molecular Probes) at room temperature for 30 min. Sections were then rinsed three times in PBS for 5 min each and then cleaned for Fluromount-G (Southern Biotechnology) mounting using dry wipers, and covered with cover slips (#1.5) for slide protection. The slides were then imaged using a confocal microscope. As this study focused on cell organizations, we decided to use the CD31 marker to enhance identification of the endothelial structures. This marker was chosen as it was expressed in a uniform manner for both HUVECs and HAMECs, compared to another commonly used marker (vWF) as shown in Additional file 2.

### Scaffold imaging

Images were captured using a Leica™ TCS LSI super-zoom macroconfocal microscope. The depth of imaging was 300–500 microns, and at least 20 Z-stacks. The seeded side of the scaffold was imaged. A X5 apochromatic macro-objective was used for whole-mount construct imaging. After completing whole-mount imaging, the scaffolds were sliced, as described above, stained with immunofluorescently labeled antibodies and imaged via an inverted microscope (Zeiss MTB2004, Carl Zeiss, Germany). The images were captured using a CCD camera (AxioCam MRm; ×40 lens).

### Vessel network development determination

Several regions of vasculature were randomly chosen for imaging within each scaffold. The various stages of development were categorized as: single cells, cell groups, a moderately developed vessel network, or a fully developed vessel network. Each development stage was then graded manually as either 25 %, 50 %, 75 % or 100 % to indicate its level within the image, thus providing an indication between the vessel development stage and given percentage value.

### Vessel length quantification using AngioTool

The confocal microscope images were uploaded to AngioTool (AngioTool®) [20], which is a program capable of detecting, marking and scoring the average vessel length (mm). The software marks all detected elements in red, junctions in blue and vessel borders in yellow.

### Vessel network complexity evaluation

Complexity [21] of the formed vessel network within the 3D scaffolds was determined using MATLAB (MATLAB^©^, MathWorks). The confocal microscope image was converted to a binary image using the threshold cut. Noise reduction was performed using erosion followed by dilation, used to restore connectivity to connect 1-pixel neighboring vessels (all morphological operations were done using bwmorph). The area and perimeter of the element detected within the image were then determined using regionprops. For each element with an area exceeding 30 pixels, a complexity factor was calculated as follows:

$$ \begin{array}{c}\hfill Complexity\kern0.5em  factor\kern0.5em =\kern0.5em \frac{4\pi A}{P^2}\kern0.1em ;\kern0.5em 0<\kern0.5em  complexity\kern0.5em  factor\kern0.5em <\kern0.5em 1;\hfill \end{array} $$

Where A is area and P is perimeter. The complexity factor is 1 for a perfect circle, and very low for complex branched structure. The various complexities per image were marked by coloring the elements according to their complexity factor, using the following scale: single cells with a rounded shape fell within the high complexity range of 0.55–1 and were marked in blue, whereas elongated but non-complex shapes received complexity values of 0.3–0.55 and were marked in yellow. Optimal vessel structures, comprised of an elongated and complex vessel network, fell within the low complexity range of 0–0.3 and were marked in red. Gray elements represent elements larger than noise size particles.

### Vessel orientation determination

Whole-mount-stained scaffolds were imaged by confocal microscopy. As aligned vessels were distributed at different angles within the scaffold, images of different regions were included in the vessel orientation analysis.

A MATLAB algorithm based on Hough-transform was used to detect lines within the image and to estimate their orientation. Histograms of line angle distributions, at 30-degree intervals ranging from −90 to 90 degrees, were generated.

A random region of detected lines was imaged in higher magnification (×5). Following that, MATLAB analysis generated a histogram of element orientation distribution. In order to determine whether the vessels demonstrated a specific direction or orientation, the maximal number of elements per direction was divided by the total number of elements in the image. Calculations are presented in histograms and compared for all tested cell pairs.

### Vessel maturity evaluation by cell marker follow-up

Whole-mount scaffold sections were labeled with antibodies against human CD31 (presented in green) and human α-SMA (presented in red), as described above, and imaged with an inverted microscope (Zeiss MTB2004, Carl Zeiss, Germany), using a 40× objective to identify vessel lumens. Several regions within each section were randomly selected. Image J [22] was used to observe the complete image. Lumens with a diameter larger than 35 microns, that may represent rounded structures that cover the scaffold pores, were excluded from the analysis. After locating the lumens for analysis, the image was split into two separate channels: red and green. The green-labeled vessel perimeter and then the red-labeled vessel fragments were manually marked within the software. The percentage of the summed length of red fragment versus the summed lumen perimeters was calculated. Each image included an average of 2–3 lumens; the average percentage of α-SMA staining with respect to the total lumen perimeter was calculated for each image.

### Statistical analysis

Presented data include the mean ± standard error of the mean. Two-way analysis of variance (ANOVA) was performed to examine the influence of two independent categorical variables followed by Bonferroni’s multiple comparison tests. Results were considered significant for *p* < 0.05. Statistical analyses were performed using a computerized statistical program (GraphPad Software, Inc.).

## Results

### Vessel-like network development on PLLA/PLGA constructs

**Fig. 1:**
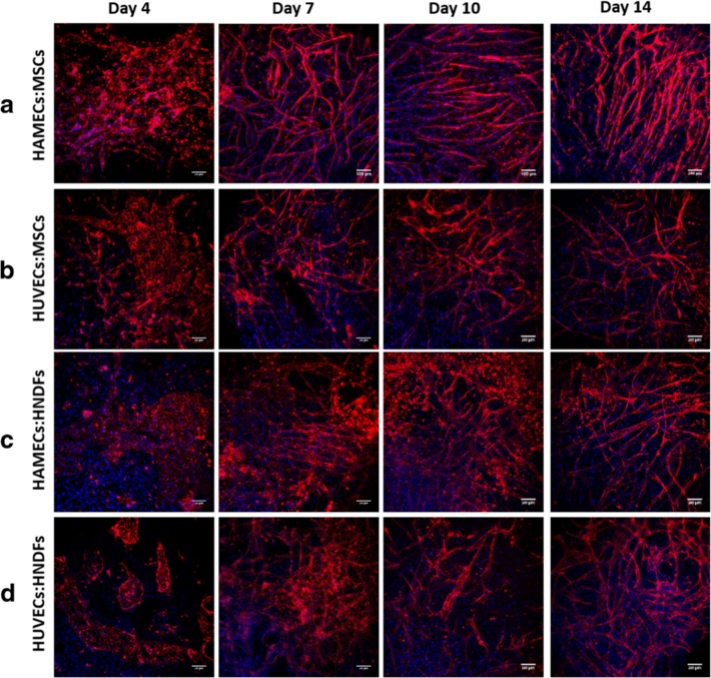
In vitro vessel-like network formation. Confocal images (scale bars = 1000 μm) of whole-mount PLLA/PLGA scaffolds embedded with **a** HAMECs and MSCs, **b** HUVECs and MSCs, **c** HAMECs and HNDFs or **d** HUVECs and HNDFs and stained for CD31 (*red*) and nuclei (*blue*), on days 4, 7, 10 and 14 post-seeding. *HAMEC* Human adipose microvascular endothelial cell, *HNDF* Human neonatal dermal fibroblast, *HUVEC* Human umbilical vein endothelial cell, *MSC* Mesenchymal stem cell

In an effort to comprehend how vessel networks develop during in vitro culture, fluorescently stained HAMECs and HUVECs grown in various co-culture combinations on 3D PLLA/PLGA matrices were tracked over a 14-day period using confocal microscopy. After 4 days in culture, cells in HAMEC:MSC (Fig. 1a) combinations began to form elongated structures, while those in the HUVEC:MSC (Fig. 1b), HAMEC:HNDF (Fig. 1c) and HUVEC:HNDF (Fig. 1d) combinations formed clusters. Well-developed vessel-like networks of HAMECs:MSCs were already observed on day 7 (Fig. 1a), and were maintained throughout the remainder of the experiment. On day 7, HUVECs:MSCs began to form elongated structures, which failed to prosper (Fig. 1b). HAMECs:HNDFs and HUVECs:HNDFs successfully formed fully developed networks on day 14 only (Fig. 1c and Fig. 1d).

### Vessel network development analysis

**Fig. 2:**
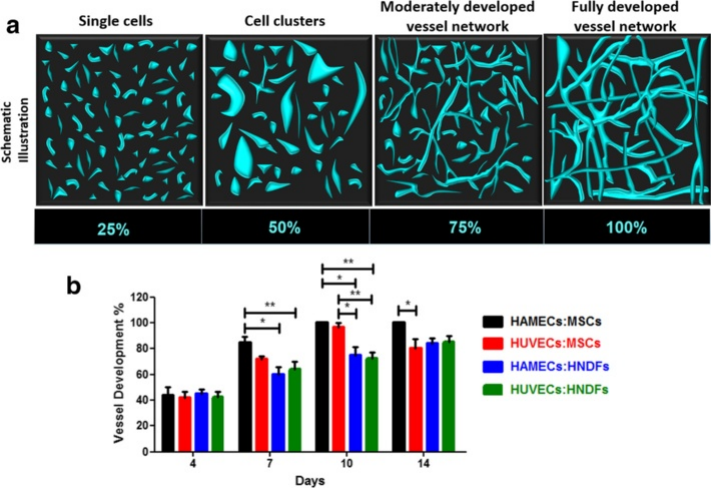
Vessel-like network development in vitro. **a** Schematic illustration of four stages of developing vessels: single cells, clusters, initial vessels and advanced vessels. The stages for development were manually scored using a percentage scale (0–100 %), where a higher percentage was given to more developed vessel-like networks. **b** Comparison of vessel development in scaffolds bearing different cell combinations, assessed at different time points in culture. Two-way ANOVA, *p* value = 0.0297, *n* ≥ 4. *p < 0.05, **p < 0.01. *HAMEC* Human adipose microvascular endothelial cell, *HNDF* Human neonatal dermal fibroblast, *HUVEC* Human umbilical vein endothelial cell, *MSC* Mesenchymal stem cell

The prevalence of vessel development stages, depicted in Fig. 2a, in randomly imaged segments was determined. By day 7, the HAMEC:MSC combination yielded organized and established vessel networks lacking cell clusters and separate single cells (Fig. 2b), while other groups formed moderately developed networks (*p* < 0.05) that comprised both clusters and many single cells, as well as initial vessel structures surrounded by single cells. In addition, aside from the HAMEC:MSC combination, which demonstrated and maintained complex vessel structure from early stages, vessel maturity gradually increased in all groups (Fig. 2b). On days 7, 10 and 14, development stages of vessel networks formed by the HAMEC:MSC combination were significantly high (80–100 %), when compared to other group combinations (60–90 %; *p* = 0.0294) (Fig. 2b).

### Vessel length quantification

**Fig. 3:**
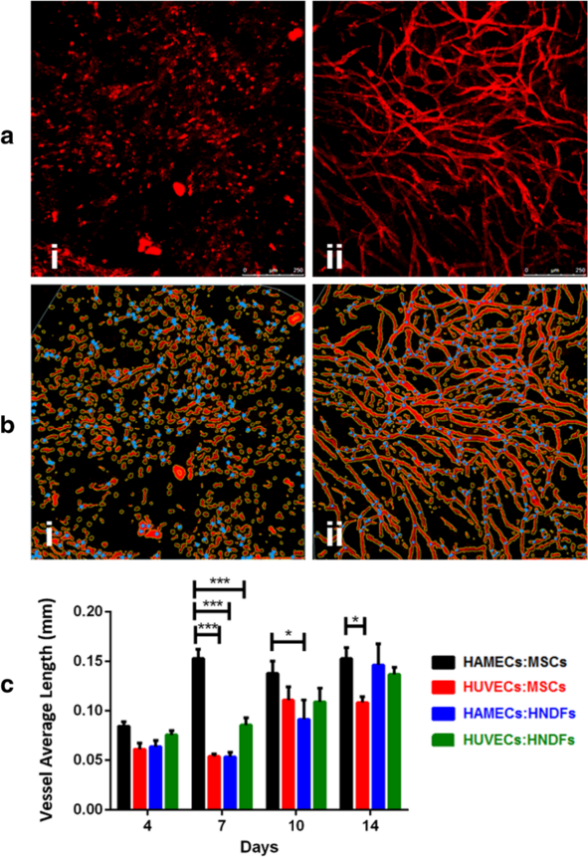
Representative images of AngioTool analysis and average vessel length determination. **a** Representative images (scale bars = 250 μm) of *i* single cells and *ii* developed vessel-network embedded within scaffolds, as captured through a confocal microscope. **b** The AngioTool program marks the *i* single cells (*red*) or *ii* developed vessels (*red*), junctions (*blue*) and vessel borders (*yellow*). **c** Average vessel length for different cell combinations was then determined. Two-way ANOVA, *p* value = 0.016, *n* ≥ 3. *p < 0.05, ***p < 0.001. *HAMEC* Human adipose microvascular endothelial cell, *HNDF* Human neonatal dermal fibroblast, *HUVEC* Human umbilical vein endothelial cell, *MSC* Mesenchymal stem cell

In vitro vessel formation on PLLA/PLGA constructs imaged by a confocal microscope (Fig. 3a) was then analyzed using a computational tool for quantitative analysis of vascular network parameters (AngioTool [20]) (Fig. 3b). All cell combinations developed vessels of a mean length of ~55 microns 4 days post-seeding; however, within 7 days the HAMEC:MSC group generated vessels of an average length of 150 microns, while other cell combinations maintained a length of ~55 microns (*p* < 0.001; Fig. 3c). HAMEC:MSC vessel length was maintained at 150 microns for up to day 14 (Fig. 3c). Other cell combinations developed gradually from a length of ~55 microns on day 7, to ~100 microns on day 10 and finally reached ~100–150 microns 14 days post-seeding (Fig. 3c).

### Vessel network complexity

**Fig. 4:**
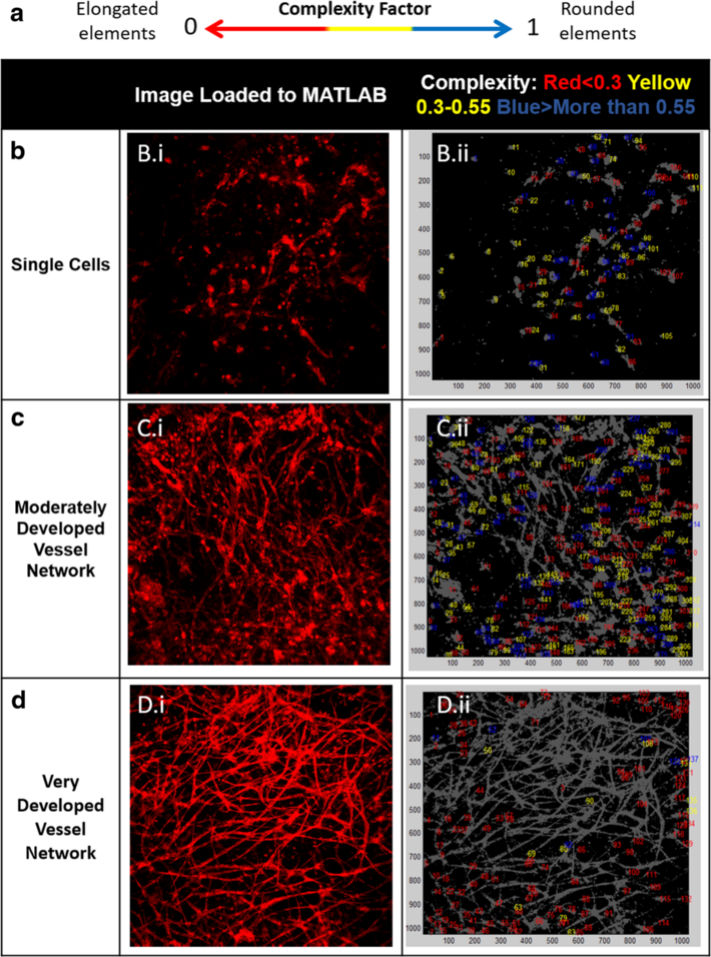
MATLAB analysis of vessel network complexity. MATLAB analysis of confocal images (scale bars = 250 μm) (*i*) generated new images (*ii*) showing vascular network elements (*grey*), upon which differently colored complexity factor values are presented. These values were divided into three broad ranges: **a** *red* <0.3, *yellow* 0.3–0.55 and *blue* >0.55. **b** Blue elements represent rounded single cells and yellow elements the more elongated structures. **c** Blue and yellow elements represent rounded cells and elongated structures, respectively, combined with more red elements, representing long and complex vessels. **d**  Dominance of red elements representing long and complex vessels and less yellow and blue elements for rounded shapes

**Fig. 5:**
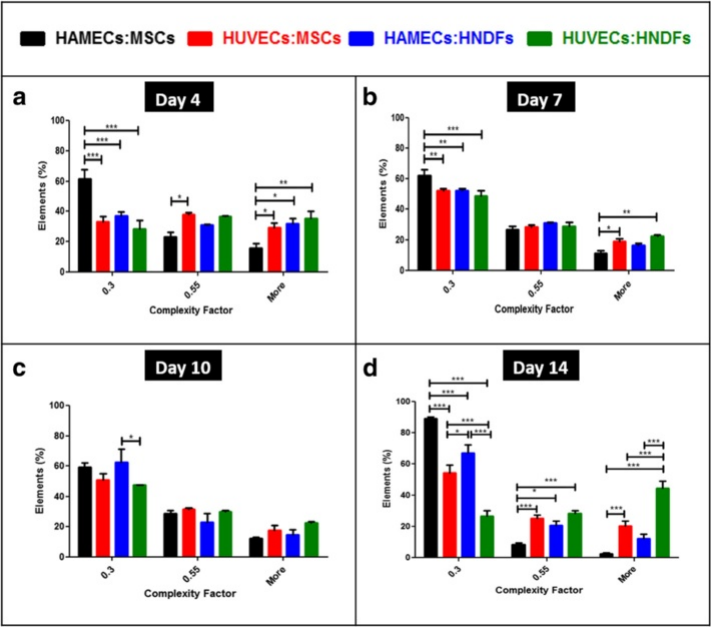
Distribution of complexity factor ranges for the different cell pairs. The complexity factor of networks within cell-embedded scaffolds was determined on days 4, 7, 10 and 14 post-seeding. The distribution of complexity factors per cell pair and per day of analysis is presented. **a** Day 4 (*p* < 0.001. **b** Day 7 (*p* = 0.003). **c** Day 10 (*p* = 0.055). **d** Day 14 (*p* < 0.001). Two-way ANOVA, *n* ≥ 3. *p < 0.05, **p < 0.01, ***p < 0.001. *HAMEC* Human adipose microvascular endothelial cell, *HNDF* Human neonatal dermal fibroblast, *HUVEC* Human umbilical vein endothelial cell, *MSC* Mesenchymal stem cell

Network complexity was then evaluated with a self-written algorithm as demonstrated in Fig. 4. On day 4, the HAMECs:MSCs cell combination formed more elongated than rounded shapes, when compared to other cell combinations (60 % vs. 40 %, *p* < 0.001; Fig. 5a). In contrast, the other cell pairs showed no statistically significant differences between the three complexity value ranges. On day 7, the HAMEC:MSC combination showed more elongated shapes in comparison to other groups (60 % vs. 50 %, *p* = 0.003; Fig. 5b). On day 10, no statistically significant differences in complexity values were observed between the tested groups (~60 % for all groups, *p* = 0.055; Fig. 5c). On day 14, ~90 % of the HAMEC:MSC-embedded scaffold elements exhibited elongated shapes, while complexity values equal to those recorded on day 10 were measured for the HUVEC:MSC and HAMEC:HNDF groups on day 14 (*p* < 0.001). The HUVEC:HNDF group exhibited more rounded than elongated shapes on day 14 (Fig. 5d).

### In vitro vessel alignment as an indication of vessel maturity

**Fig. 6:**
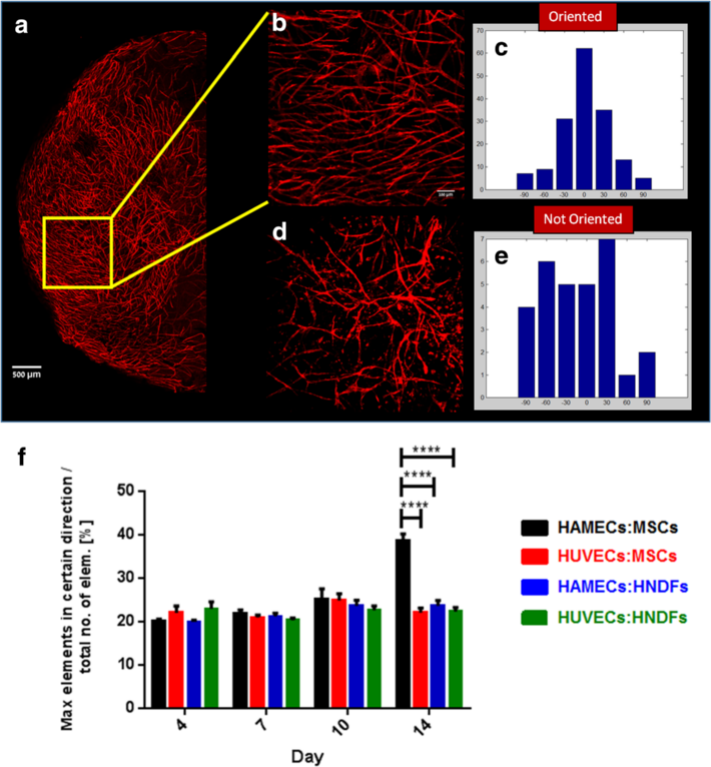
Vessel alignment as an indication of vessel maturity. **a** Representative image of half of a scaffold showing different regions with orientated vessels (scale bar = 500 μm). **b** Magnified image of vessels orientated in a specific direction (scale bar = 100 μm) and **c** a histogram showing the distribution of the elements orientation in image **b** (represented by different angles). **d** A magnified image of non-oriented vessels (scale bar = 100 μm) and **e** a histogram showing the distribution of the elements orientation in image **d** (represented by different angles). **f** Statistical analysis of the number of elements in a given direction versus the total number of elements (%) in each field of view. Two-way ANOVA, Bonferroni’s multiple comparisons test, *n* ≥ 4. ****p < 0.0001. *HAMEC* Human adipose microvascular endothelial cell, *HNDF* Human neonatal dermal fibroblast, *HUVEC* Human umbilical vein endothelial cell, *MSC* Mesenchymal stem cell

One phenomenon that has been seen in the present study of whole-stained co-cultured scaffolds during vessel development over time is the ability of vessels to spontaneously align with proximal vessels in the scaffolds. Vessel-orientation directions were distributed randomly within the scaffold; thus, several random segment were imaged and assessed. Up to day 14, orientation preference was only observed among 20 % of the detected elements in all cell pairs (Fig. 6f). However, on day 14 the HAMEC:MSC combination showed an orientation preference among 40 % of the detected elements per field of view (Fig. 6f), while other cell combinations only showed an orientation preference among 20 % of elements (*p* < 0.0001; Fig. 6f).

### α-SMA expression as an indicator for vessel maturity

**Fig. 7:**
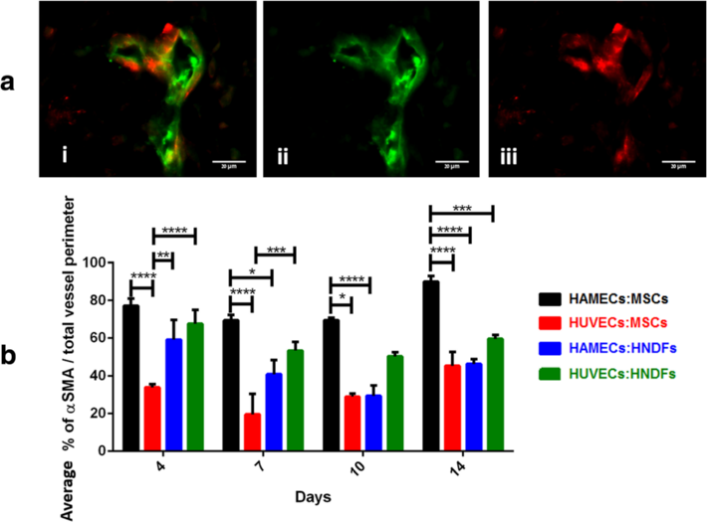
Evaluation of vessel maturity using cell markers. **a** Paraffin-embedded sections (5 μm) of cell-embedded PLLA/PLGA scaffolds were fluorescently labeled for *i* α-SMA (*red*) and *ii* CD31 (*green*) (scale bars = 20 μm). *iii* A merged image is presented for α-SMA and CD31 staining (scale bar = 20 μm). **b** Statistical analysis of α-SMA coverage out of the overall lumen perimeter, determined by CD31 staining, was performed for vessels under 35 μm in diameter. Two-way ANOVA, Tukey’s multiple comparisons test, *n* ≥ 4. *p < 0.05, **p < 0.01, ***p < 0.001, ****p < 0.0001. *α-SMA* Alpha-smooth muscle actin, *HAMEC* Human adipose microvascular endothelial cell, *HNDF* Human neonatal dermal fibroblast, *HUVEC* Human umbilical vein endothelial cell, *MSC* Mesenchymal stem cell

To assess vessel maturity, α-SMA and CD31 biomarkers were labeled and analyzed under a fluorescence microscope (Fig. 7a). From day 4 until day 14, in the HAMEC:MSC group, 80 % of the total perimeter of detected lumens were α-SMA-positive, while other cell combination expressed significantly less α-SMA (40–60 %; *p* < 0.001) (Fig. 7b). In addition, the HUVEC:MSC group showed the lowest relative α-SMA expression on all tested days (Fig. 7b) when compared to other cell combinations. The HUVEC:HNDF group followed the HAMEC:MSC group in its relative α-SMA expression levels (Fig. 7b).

## Discussion

As bioengineered tissues require mature blood vessels for optimal functionality and integration in vivo [3, 15], understanding the vessel formation process is important to promote and optimize vessel creation in vitro. The present study demonstrated how the use of adipose-derived stem cells combined with microvascular ECs enhances and upgrades vessel network formation in vitro. This combination led to rapid development of vasculature with highly complex networks that expressed mature vessel biomarkers which even became aligned as they developed.

Numerous studies have shown that adipose-derived microvascular ECs comprise a rich source of pro-angiogenic factors, such as vascular endothelial growth factor and basic fibroblast growth factor [14, 23, 24]. In addition, compared to macrovessels (aortic ECs or umbilical vein ECs), microvascular ECs secrete more angiogenic factors as a result of the physiological nature of adipose tissue [14, 25]. Addition of MSCs has been suggested to improve vascularization by secretion of more angiogenic markers than fibroblasts [26–28]. The presented findings further strengthen the well-established notion regarding MSC-driven promotion of vascular formation within 3D scaffolds, as well as the ability of microvascular ECs to effectively form vessels.

In earlier studies with a HUVEC:HNDF combination, cell clusters formed which then continuously sprouted and formed microvessels [4, 29]. In contrast, the HAMEC:MSC combination studied here formed microvessels within the first few days in culture, without cluster organization, which then rapidly formed vascular networks. These observations suggest a unique role played by adipose MSCs in facilitating and promoting angiogenic potential beyond those observed with HNDFs [30]. In addition, compared to macrovessels (aortic ECs or umbilical vein ECs), microvascular ECs secrete more angiogenic factors as a result of the physiological nature of adipose tissue [14, 25, 31]. These differences were manifested here in the significantly different time course of vascular development and maturation in samples containing HAMECs versus HUVECs.

Within 7 days of seeding, the HAMEC:MSC-embedded scaffolds featured vessels of an average length of 150 microns, which corresponds to the length of the longest vascular structures measured within a heterogeneous mixture of adipose-derived arterioles, capillaries, and venules [32, 33]. In contrast, the other cell combinations reached this length only 14 days post-seeding. We suggest that vessels formed in vitro can reach natural dimensions and characteristics that can be beneficial for future in vivo and clinical studies.

Furthermore, vessel formation and elongation in the HAMEC:MSC group occurred within 4 days of culture and reached maximal complexity on day 14. Other cell combinations did not display network complex as high as the HAMEC:MSC group. In addition, a larger number of oriented vessel segments were observed in the HAMEC:MSC scaffolds, indicating vessel maturation [15]. As vessel alignment typically occurs in vitro following external strain and stress cues on the cell-seeded scaffold [34–36] but formed spontaneously in the present study, we suggest further investigation of this process. In order to further investigate vessel development, we aimed to track an accessible biological cell marker indicative of their maturity. Newly formed vessels are initially created by the ECs [37]. However, as vessels mature the presence of α-SMA increases due to the recruitment of supporting cells such as HNDFs or MSCs [38, 39]. As maturation continues, vessel walls become rich either in smooth muscle cells, myofibroblasts or pericytes that express α-SMA [34, 40]. Therefore, α-SMA has become an accepted marker of vessel maturity. The presented data demonstrate that between days 4–14 in culture, the HAMEC:MSC-embedded scaffolds expressed more α-SMA than any other cell combination.

## Conclusions

Taken together, adipose-derived stem cells combined with microvascular ECs demonstrate powerful angiogenic and vasculogenic capabilities. We assume that highly complex vessel networks within engineered tissues will improve integration within the host tissue. Moreover, EC-MSC-embedded scaffolds may present an appropriate model for studying angiogenesis under physiologically relevant conditions. In addition, engineering of vascularized scaffolds can be effectively upgraded by integrating easily harvestable human adipose cells that include MSCs and microvascular ECs which can be advantageous in many in vivo applications toward treatment of tissue loss or defects requiring rapid vasculogenesis, and in design of effective tissue engineering solutions.

## Additional files

Additional file 1: Figure S1. Whole PLLA/PLGA scaffolds were cut perpendicularly to their horizontal plane into 5-micron sections. (DOCX 60 kb) Additional file 2: Figure S2. CD31 vs. vWF distribution in ECs of different origins. Paraffin-embedded sections (5 μm) of HAMECs:MSCs and HUVECs:HNDFs cultured for 7 days on PLLA/PLGA scaffolds, were stained for **Ai** DAPI (*blue*), **Aii** vWF (*red*) and **Aiii** CD31 (*green*) (scale bars = 50 μm). **Aiv** A merged image of the DAPI, vWF and CD31 staining is presented (Scale bar = 50 μm). Lumens are indicated with *white arrows*. (DOCX 209 kb)

## Abbreviations

3D: Three-dimensional
ANOVA: Analysis of variance
BSA: Bovine serum albumin
DMEM: Dulbecco’s modified Eagle’s medium
ECs: Endothelial cells
FBS: Fetal bovine serum
HAMEC: Human adipose microvascular endothelial cell
HNDF: Human neonatal dermal fibroblast
HUVEC: Human umbilical vein endothelial cell
MSC: Mesenchymal stem cell
PBS: Phosphate-buffered saline
PLGA: Poly-lactic-co-glycolic acid
PLLA: Poly-L-lactic acid
α-SMA: Alpha-smooth muscle actin
