# Supplementary material for: Adipose-derived endothelial and mesenchymal stem cells enhance vascular network formation on three-dimensional constructs in vitro
Source: Stem Cell Res Ther. 2016 Jan 11;7:5. doi: 10.1186/s13287-015-0251-6 (PMC4709933; doi:10.1186/s13287-015-0251-6)
Supplement: Additional file 1: Figure S1. — Whole PLLA/PLGA scaffolds were cut perpendicularly to their horizontal plane into 5-micron sections. (DOCX 60 kb) [file 13287_2015_251_MOESM1_ESM.docx]

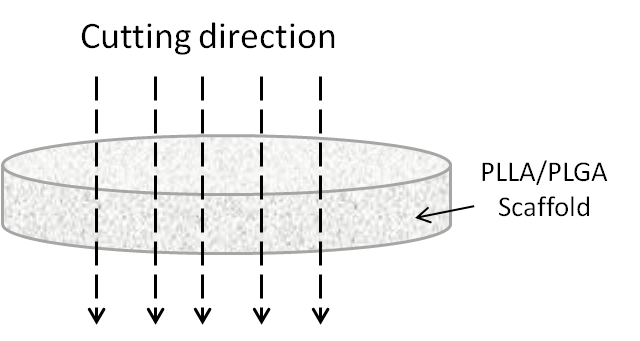


**Additional Figure 1**. Whole PLLA/PLGA scaffolds were cut perpendicularly to their horizontal plane, to 5-micron sections.
