# Supplementary material for: Adipose-derived endothelial and mesenchymal stem cells enhance vascular network formation on three-dimensional constructs in vitro
Source: Stem Cell Res Ther. 2016 Jan 11;7:5. doi: 10.1186/s13287-015-0251-6 (PMC4709933; doi:10.1186/s13287-015-0251-6)
Supplement: Additional file 2: Figure S2. — CD31 vs. vWF distribution in ECs of different origins. Paraffin-embedded sections (5 μm) of HAMECs:MSCs and HUVECs:HNDFs cultured for 7 days on PLLA/PLGA scaffolds, were stained for Ai DAPI (blue), Aii vWF (red) and Aiii CD31 (green) (scale bars = 50 μm). Aiv A merged image of the DAPI, vWF and CD31 staining is presented (Scale bar = 50 μm). Lumens are indicated with white arrows. (DOCX 209 kb) [file 13287_2015_251_MOESM2_ESM.docx]

| **(A.i) DAPI** | **(A.ii) CD31** | **(A.iii) vWF** | **(A.iv) MERGE** |
| --- | --- | --- | --- |

**
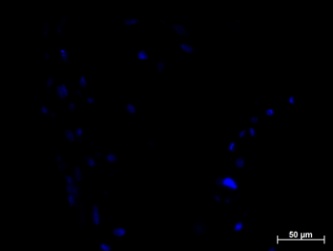

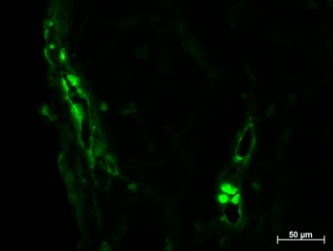

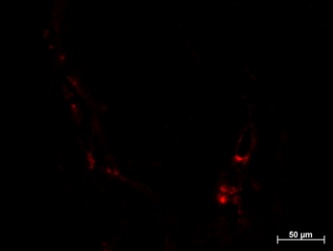

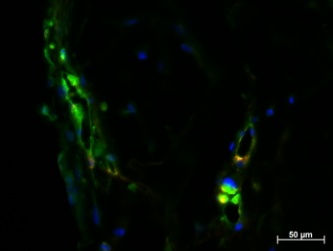
**

**HAMECs:MSCs**

Lumens

Lumens

**
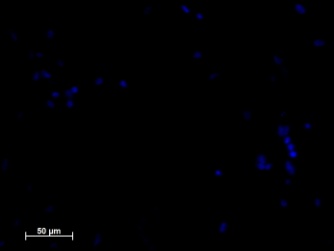

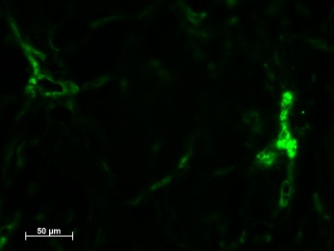

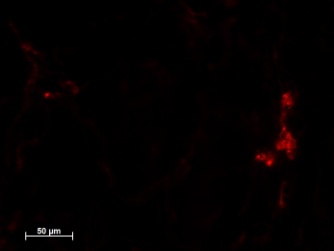

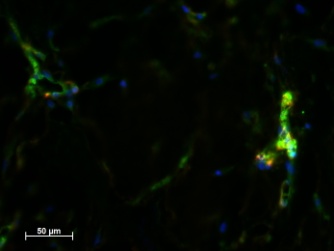
**

Lumens

|  |  |  |  |
| --- | --- | --- | --- |

**
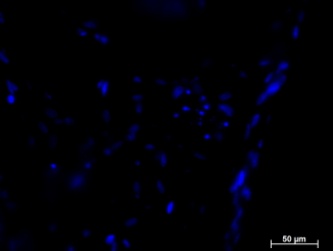

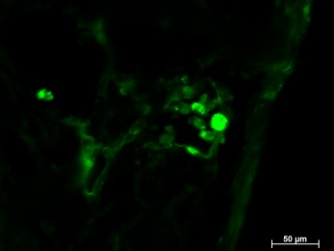

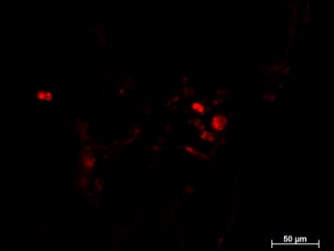

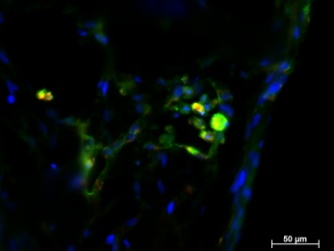
**

**HUVECs:HNDFs**

Lumens

Lumens

**
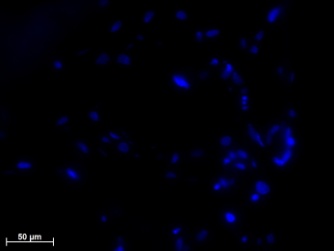

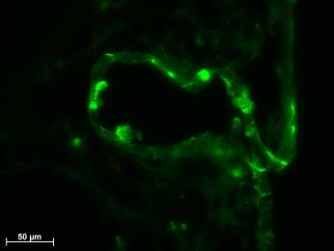

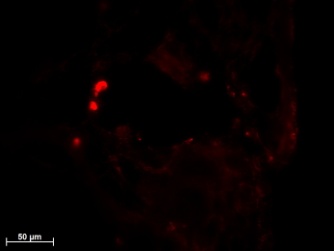

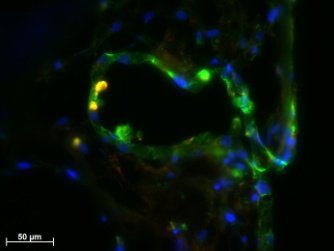
**

Lumens

**Additional Figure 2**. **CD31 vs. vWF distribution in ECs of different origins.** (A.i) Paraffin-embedded sections (5µm) of HAMECs:MSCs and HUVECs:HNDFs cultured for 7 days on PLLA/PLGA scaffolds, were stained for DAPI (blue); (A.ii) vWF (Red) and (A.iii) CD31 (Green) (Bar=50μm). (A.iv) A merged image of the DAPI, vWF and CD31 staining is presented (Bar=50μm). Lumens are pointed with white arrows.
